# Time Pressure Increases Cooperation in Competitively Framed Social Dilemmas

**DOI:** 10.1371/journal.pone.0115756

**Published:** 2014-12-31

**Authors:** Jeremy Cone, David G. Rand

**Affiliations:** Yale University, New Haven, Connecticut, United States of America; Center of nonlinear, China

## Abstract

What makes people willing to pay costs to benefit others? Does such cooperation require effortful self-control, or do automatic, intuitive processes favor cooperation? Time pressure has been shown to increase cooperative behavior in Public Goods Games, implying a predisposition towards cooperation. Consistent with the hypothesis that this predisposition results from the fact that cooperation is typically advantageous outside the lab, it has further been shown that the time pressure effect is undermined by prior experience playing lab games (where selfishness is the more advantageous strategy). Furthermore, a recent study found that time pressure increases cooperation even in a game framed as a competition, suggesting that the time pressure effect is not the result of social norm compliance. Here, we successfully replicate these findings, again observing a positive effect of time pressure on cooperation in a competitively framed game, but not when using the standard cooperative framing. These results suggest that participants' intuitions favor cooperation rather than norm compliance, and also that simply changing the framing of the Public Goods Game is enough to make it appear novel to participants and thus to restore the time pressure effect.

## Introduction

Cooperation is, at once, both an essential feature of human social life and an enduring puzzle: why would anyone incur the personal costs that cooperative behavior demands in order to benefit the group when he or she could just as easily behave selfishly and hope to reap the rewards of others' prosociality? As such, the evolution and maintenance of cooperation are topics of major interest across the social and biological sciences [1]–[32].

Research has attempted to better understand the cognitive mechanics of cooperative decision-making by appealing to dual process models of judgment. These models posit the existence of two qualitatively distinct modes of thought: one that is relatively automatic, rapid, spontaneous, holistic, and intuitive, and another that is relatively controlled, slow, sequential, deliberative and rational [33]–[39]. Through the lens of this dual process perspective, a key question on the nature of cooperative decision-making concerns the extent to which individuals possess an intuition to be selfish that is only overridden through deliberative efforts to be prosocial, or, instead, possess an intuition towards prosociality that is overridden by deliberative selfishness.

To assess these two possibilities, a number of recent studies have attempted to experimentally manipulate the extent to which intuitive or rational processes are engaged during decision-making in economic cooperation games. It has been found that time pressure [40]–[43], cognitive load [44]–[46], conceptual priming of intuition [40], deciding about present rather than future allocations of money [47], [48], and disruption of the right lateral prefrontal cortex [49] can increase participants' willingness to pay money to benefit others in both unilateral and multilateral cooperation games. Other studies find null effects of some of these manipulations [50]–[53], suggesting the presence of important moderators. (Reaction time *correlation* studies yield conflicting results [40], [54]–[58], but this is likely due to the fact that decisions involving conflict take longer, regardless of the extent to which intuitive versus deliberative processes are invoked [59]; furthermore, ego depletion seems to result in opposite effects on Dictator Game prosociality compared to the other manipulations of cognitive processing [60]–[63] suggesting that in this context, depletion may be both reducing self-control *and* changing participants' intuitive preferences.)

Further evidence for an intuitive predisposition towards cooperation comes from the finding that participants treat neutrally framed games the same as cooperatively framed games [64]; that previous play of long versus short repeated games spills over into subsequent one-shot games, but only for participants who rely on heuristics [65]; that people with low self-control are more likely to sacrifice to benefit their romantic partners [66]; that people who risk their lives to save strangers overwhelmingly describe their decision processes as automatic and intuitive [67]; that text analysis of participants' descriptions of their decision process during economic games finds that positive emotion predicts cooperation while inhibition predicts selfishness [68]; and the cooperative choices involve less conflict than non-cooperative choices in mouse-tracking studies [69].

### Theoretical Motivation

To explain this overall relationship between deliberation and selfishness, and to predict specific moderators, the Social Heuristics Hypothesis (SHH) has been proposed [41]. The SHH adds an explicitly dual process framework to theories of cultural evolution, norm internalization and spillover effects [8], [70]–[75]. It posits that cooperative decision-making is guided by heuristic strategies that have generally been successful in one's previous social interactions and have, over time, become internalized and automatically applied to social interactions that resemble situations one has encountered in the past. When one encounters a new or atypical social situation that is unlike previous experience, one generally tends to rely on these heuristics as an intuitive default response. However, through additional deliberation about the details of the situation, one can override this heuristic response and arrive at a response that is more tailored to the current interaction. Thus, misapplication and over-generalization of heuristics is at the heart of the SHH.

An important prediction that stems from this over-generalization is that the effects of promoting intuition should be moderated by one's past experience. Because cooperative decision-making in one's daily life often involves repeated interactions with others in which reputation or the threat of sanctions is an important consideration, intuitions should generally favor cooperation because this is the payoff-maximizing strategy. However, not everyone acquires such experience in their daily lives, and those individuals that report having less general trust of their interaction partners in their daily lives have been shown to exhibit less of a tendency to cooperate when induced to rely more heavily on their intuitions [40], [42].

Moreover, even if individuals more generally have cooperative interactions in their daily lives, they may nonetheless have a great deal of exposure to situations or contexts in which cooperation is not the payoff-maximizing strategy, thus leading them to be less inclined to trust their intuitions in these cases. That is, experience with settings where one's typically advantageous response is non-optimal should lead to a reduction in the spillover effects that the SHH argues drive the intuitive cooperation effect. In support of this contention, people's self-reported experience with economic games—games in which the selfish strategy is typically payoff-maximizing—tend to exhibit less of an effect of promoting intuition on their cooperative decision-making [40]–[42]. Moreover, in a longitudinal analysis of the effects of time pressure in economic games conducted online on Mechanical Turk, intuitions to cooperate steadily declined over a two-year period, suggesting that as economic games became more popular on MTurk and as participants acquired greater experience with them, they exhibited less of a tendency to trust intuitions to cooperate [41].

If this effect of prior experience with economic games is indeed driven by a learned suppression of spillover effects in the context of familiar game paradigms, then it should be possible to re-induce the intuitive cooperation effect by modifying the paradigm such that it once again appears novel. One way to achieve this may be to change the way in which the cooperative decision is framed. The results of a recent study [43] (hereafter RNW) are consistent with this suggestion. In their second study, RNW applied time pressure or delay to cooperation games while also manipulating how the cooperative decision was framed to participants (RNW's first study crossed time pressure with an ingroup/outgroup manipulation). In the competitive frame condition, the interaction was described as a competition with other competitors, with a winner being declared at the end of the game on the basis of their earned payoffs. In the cooperative frame condition, the game used the language typical of many cooperation experiments (inspired by the seminal work of [76]) in which the game was described as a decision about how much to contribute to a common project. Previous research has suggested that such differences in framing can have strong effects on participants' overall levels of cooperation [64], [77]. RNW asked whether, in addition to having a main effect on cooperation, the framing manipulation would moderate the effects of time pressure on participants' choices in a Public Goods Game (PGG). They found main effects of context and time pressure in the predicted directions. With respect to moderation, they found a non-significant but trending interaction, such that the positive effect of time pressure on cooperation was driven primarily by those in the *competition* condition (rather than the cooperatively framed baseline typically used in PGG experiments).

This result is surprising if one believes that the effect of time pressure on cooperation is explained by increasing adherence to the social norms dictated by the situation: on this view, one would expect time pressure to lead to greater selfishness in the competitive domain and greater cooperation in the cooperative domain. If, conversely, people have a domain-general heuristic favoring cooperation (rather than norm compliance) that gets degraded by prior experience with specific settings where cooperation is not advantageous, the observed pattern should be expected: many participants on MTurk have prior experience with the standard cooperative-framed PGG instructions (undermining the intuitive cooperation effect), whereas the competitive frame is novel (leaving the intuitive cooperation effect intact). Thus, the results of RNW provide evidence that the intuitive cooperation effect is not unique to situations where a cooperative norm is projected, and provides further evidence for the experience hypothesis.

### The Present Study

Here we test whether this pattern of results is replicable. Above and beyond the general importance of replication studies, we had several additional motivations. First, although RNW found a significant positive simple effect of time pressure in the competition condition and no simple effect in the baseline, the interaction between time pressure and condition was non-significant in their data. Thus, it is difficult to definitively conclude that the novel competition frame increased the time pressure effect (as would be predicted by the experience hypothesis). Second, RNW's main analysis in their framing experiment excluded participants that failed to obey the time constraint, which can impair causal inference [50] (note that this was *not* true of RNW's Study 1, which demonstrated the robustness of the time pressure effect to interaction with in-group vs out-group members). Finally, there have been general questions raised about the replicability of the effect of time pressure on cooperation [50], [51], [78], and so further tests are valuable.

To this end, we sought to replicate RNW's framing experiment (their Study 2) using a large sample drawn from the same study population as the original study. Additionally, we aggregate these new data with those of RNW to assess the overall effect of frame and time pressure on cooperative decision-making.

## Methods

### Participants

Participants were 751 (319 women; M_age_ = 30.5) Mechanical Turk (MTurk) workers [79]–[83] who were located in the United States. They participated in exchange for a $0.50 show-up fee as well as the opportunity to earn up to an additional $1 based on their decisions in an economic game. These studies were approved by the Yale University Human Subjects Committee IRB Protocol #1307012383. All subjects provided written informed consent prior to participating, and this was approved by the Human Subjects Committee. For raw data, see Material S1.

### Procedure

#### Measure of cooperation

To assess participants' level of cooperation, they completed a one-shot four player Public Goods Game (PGG). Each participant made a decision about how much of an endowment of $0.40 they wanted to contribute to a shared public resource, in increments of $0.02. Participants were informed that any money they contributed to the shared resource would be doubled by the experimenter and distributed evenly among all four members of the group. Thus, if all participants contributed all $0.40 of their endowment, everyone would double their earnings and receive $0.80. However, if participants chose to keep their $0.40 while the other three group members contributed their earnings, they would receive $1.00, thus maximizing their total earnings. Our primary dependent measure was the amount of money contributed.

#### Framing manipulation

To manipulate participants' construal of the economic game as either cooperative or competitive, we altered the wording of the instructions by condition. In the *cooperative* condition, the game was described as a decision about how much to contribute to a common project, and the other participants were referred to as other members of the group:

“You have been randomly assigned to interact with 3 other people. All of you receive this same set of instructions. You cannot participate in this study more than once. Each person in your group is given 40 cents for this interaction (in addition to the 50 cents you received already for participating). You each decide how much of your 40 cents to keep for yourself, and how much (if any) to contribute to the group's common project (in increments of 2 units: 0, 2, 4, 6 etc). All money contributed to the common project is doubled, and then split evenly among the 4 group members. Thus, for every 2 cents contributed to the common project, each group member receives 1 cent. If everyone contributes all of their 40 cents, everyone's money will double: each of you will earn 80 cents. But if everyone else contributes their 40 cents, while you keep your 40 cents, you will earn 100 cents, while the others will earn only 60 cents. That is because for every 2 cents you contribute, you get only 1 cent back. Thus you personally lose money on contributing. The other people are REAL and will really make a decision – there is no deception in this study. Once you and the other people have chosen how much to contribute, the interaction is over. Neither you nor the other people receive any bonus other than what comes out of this interaction.”

In contrast, in the *competitive* condition, the interaction with the other participants was described as a competition with four competitors:

“You have been randomly assigned to compete with 3 other opponents. All of you receive this same set of instructions. You cannot participate in this study more than once. Each person in your group is given 40 cents for this interaction (in addition to the 50 cents you received already for participating). You each decide how much of your 40 cents to keep for yourself, and how much (if any) to contribute (in increments of 2 units: 0, 2, 4, 6 etc). All money contributed is doubled, and then split evenly among the 4 competitors. Thus, for every 2 cents contributed, each group member receives 1 cent. If everyone contributes all of their 40 cents, everyone's money will double: each of you will earn 80 cents. But if everyone else contributes their 40 cents, while you keep your 40 cents, you will earn 100 cents, while the others will earn only 60 cents. That is because for every 2 cents you contribute, you get only 1 cent back. Thus you personally lose money on contributing. Your opponents are REAL and will really make a decision – there is no deception in this study. Once you and the other people have chosen how much to contribute, the interaction is over. Neither you nor the other competitors receive any bonus other than what comes out of this interaction.”

(Note that in our experiment the competition instructions were more closely matched to the cooperative condition than the instructions used in the competitive condition in RNW.)

#### Manipulation of time constraint

We manipulated the cognitive processing of participants' cooperative decision-making by imposing a time constraint on their decision in the PGG. Following the instructions page (which was self-paced), participants moved to a new screen that differed by condition. In the *time constraint* condition, participants were asked, at the top of the screen, to make their decision as quickly as possible, and to take no longer than 10 seconds to make their choice. In the *time delay* condition, participants were asked to consider their decision very carefully and were told not to make a decision for at least 10 seconds. In both cases, participants made their decision using a slider initialized to a 50% contribution (as in [40], [43]).

A number of participants did not obey the time constraint instructions, either failing to make their decision within the allotted time in the *time constraint* condition (140 participants; 36%) or failing to wait the allotted time in the *time delay* condition (61 participants; 17%). However, the manipulation still had a substantial effect: the median decision time in the *time constraint* condition was 9 seconds, and, in the *time delay* condition, 21 seconds. We include all participants (regardless of whether they obeyed the time constraint) in our analyses in order to ensure a causal interpretation of our results (see [50]). We note, however, that our results are robust to exclusion of those that failed to follow the time constraint instructions.

#### Assessing Comprehension

We assessed comprehension with two questions that occurred after the decision (so as not to induce a reflective mindset, as per [40]'s Supplemental Study): “What level of contribution earns the highest payoff for the group as a whole?” and “What level of contribution earns the highest payoff for you personally?” 216 people (28.8%) answered at least one of these questions incorrectly (69 answered #1 wrong, 9.2%; 205 answered #2 wrong, 27.3%). As we demonstrate below, our results are robust to inclusion or exclusion of those that failed the comprehension questions.

## Results

**Figure 1:**
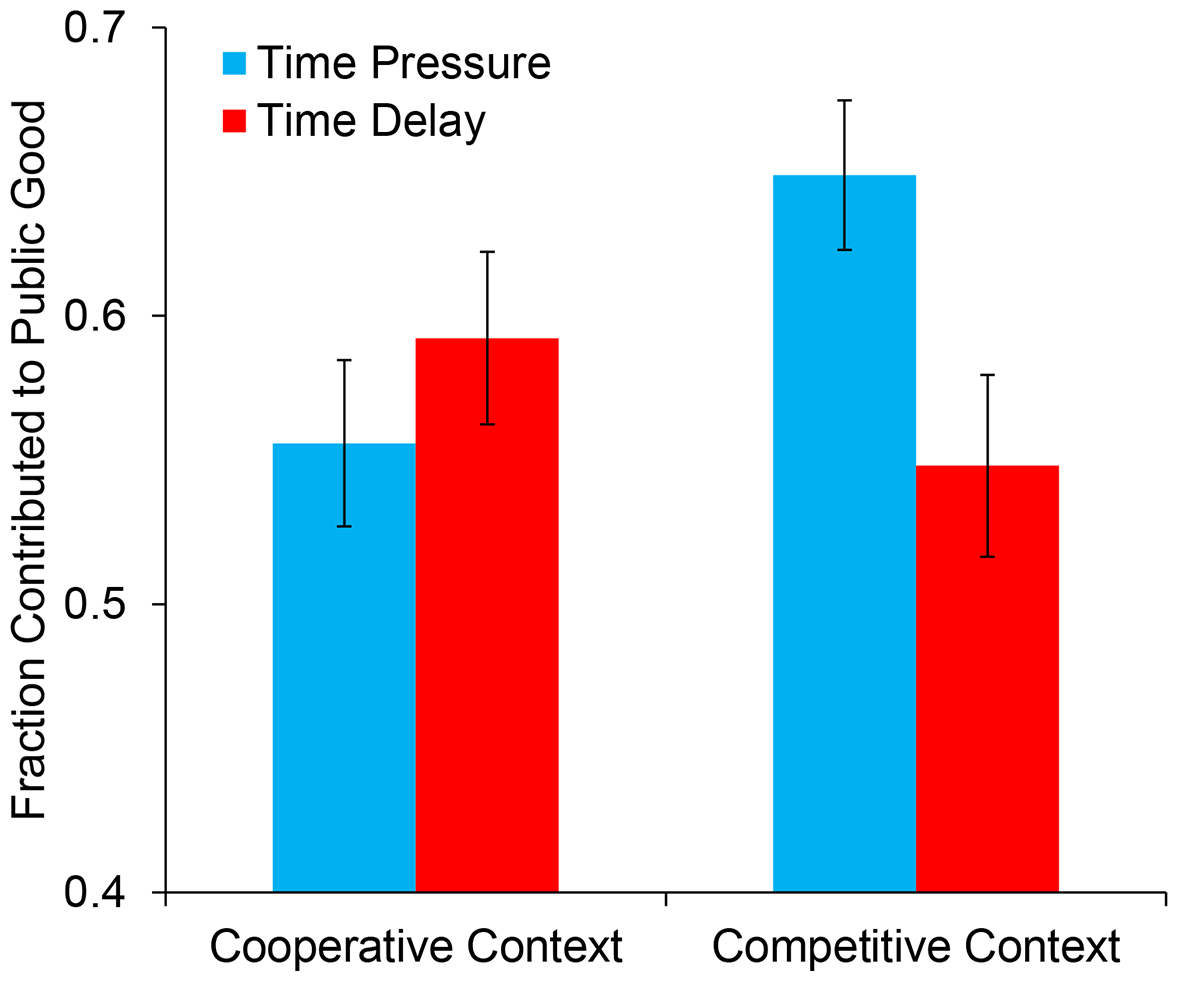
Average cooperation (% of endowment contributed to public good) by time constraint and social context in our experiment. Error bars indicate standard errors of the mean.

To assess the effects of our manipulations on participants' levels of cooperation, we submitted PGG contributions to a 2 (time constraint or time delay) ×2 (competitive or cooperative frame) analysis of variance (ANOVA). This analysis revealed a significant interaction between time constraint and contextual framing, *F*(1,747) = 5.605, *p* = .018 (see Fig. 1) (with non-comprehenders excluded: *F*(1,531) = 5.118, *p* = .024).

Examining the simple effects, we find that in the competitive framing, participants contributed significantly more to the public good when under time pressure than when forced to deliberate, *F*(1,747) = 6.075, *p* = .014 (with non-comprehenders excluded: *F*(1,531) = 6.499, *p* = .011). Conversely, there was no significant simple effect of time constraint in the cooperative framing, *F*(1,747) = .789, *p* = .375 (with non-comprehenders excluded: *F*(1,531) = .378, *p* = .539).

These results successfully replicate the simple effects found in RNW. Moreover, we find a significant interaction between time pressure and framing where RNW found only a trending interaction that was non-significant.

**Figure 2:**
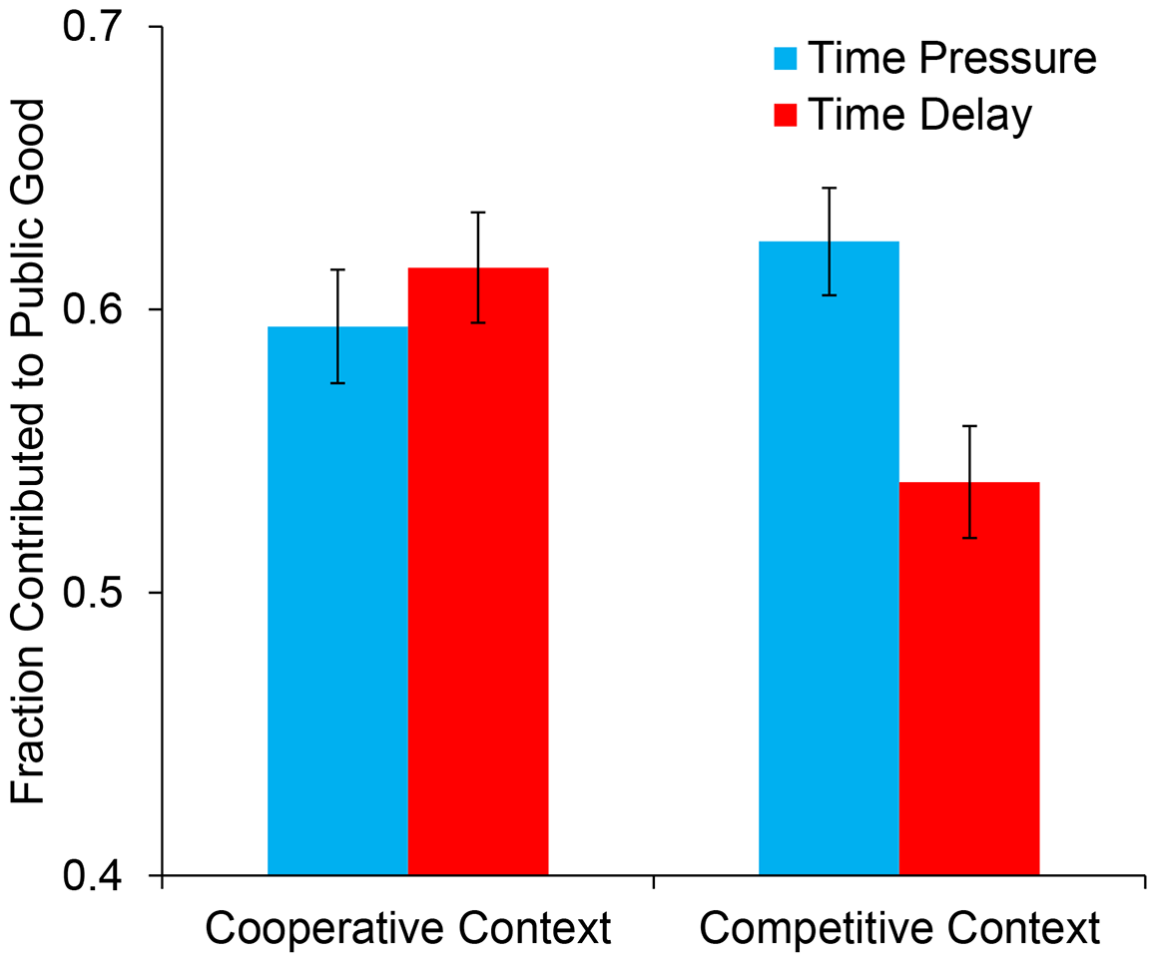
Average cooperation (% of endowment contributed to public good) by time constraint and social context when aggregating data from our experiment as well as Rand Newman & Wurzbacher (2014). Error bars indicate standard errors of the mean.

To further assess the robustness of our result, we aggregated our data with the N = 899 observations from RNW Study 2. We conducted a 2 (Study: Current or RNW) ×2 (time constraint or time delay) ×2 (competitive or cooperative frame) ANOVA. This analysis once again revealed a significant interaction between time constraint and contextual framing, *F*(1,1642) = 7.04, *p* = .008 (see Fig. 2) (with non-comprehenders excluded: *F*(1,1222 = 4.127), *p* = .042), that was, importantly, unqualified by Study, *F*(1,1642) = .699, *p* = .403 (with non-comprehenders excluded: *F*(1,1222) = 1.88, *p* = .17).

In the competitive framing, participants once again contributed significantly more to the public good when under time pressure than when forced to deliberate, *F*(1,1642) = 9.152, *p* = .003 (with non-comprehenders excluded: *F*(1,1222) = 6.848, *p* = .009). Conversely, there was no significant simple effect of time constraint in the cooperative framing, *F*(1,1642) = .533, *p* = .465 (with non-comprehenders excluded: *F*(1,1222) = .051, *p* = .821).

These results thus clarify the effect of framing on time pressure, suggesting that the interaction between frame and time pressure is significant, and that time pressure only has a positive effect on cooperation in the competitive framing in which the context served to make the decision feel less familiar.

## Discussion

Using time constraint manipulation, we replicate RNW's findings that in a Public Goods Game framed as a competition, participants were more inclined to cooperate with others when under time pressure than when asked to deliberate and think carefully about their decision. These findings thus suggest that the effects of intuitive cooperation demonstrated in previous research [40]–[43] are not merely about obeying the social norms dictated by the situation or by participants assuming that cooperation is what is implicitly expected or required of them in the experimental context. Even when the interaction was specifically framed as a competition, and the only way to win that competition was by not contributing to a public good, participants were nonetheless more inclined to cooperate when forced to make their decision quickly rather than deliberately.

These results also suggest that the intuitive cooperation effect is the result of a broad over-generalization, in that it was still observed in a setting where cooperation was less favorable. This is *inconsistent* with the idea that different social defaults exist for different scenarios, such that different frames would elicit different heuristic responses matched to the frame. On the contrary, the intuitive response in the competitively framed interaction was much *more* cooperative than the intuitive response in the cooperatively framed interaction. We also note that the time pressure effect we observe cannot be explained by increasing randomness or errors, as contribution rates are further from 50% (chance) and closer to 100% under time pressure than under time delay.

The fact that we do not observe an effect of time pressure in the baseline cooperatively framed condition is surprising from a ‘different heuristics for different situations' perspective, but consistent with recent evidence regarding the ability of prior experience with economic games to undermine cooperative intuitions [40]–[42]. It is noteworthy, then, that in the control condition in our study—under conditions that are most likely to capture the standard presentation of PGGs on MTurk and thus much more likely to be familiar to experienced turkers—we fail to find any evidence for an effect of time constraint on participants' contributions. However, it appears that only a few small changes to the wording of the instructions and presentation of the game in our competitive framing condition were enough to restore the effects of time pressure on cooperation. If this analysis is correct, it suggests that even small changes to the presentation of standard economic games (even those that might lead to an overall decrease in cooperative behavior) should result in larger effects of time pressure on decisions to cooperate.

## Supporting Information

S1 File (CSV)
